# Evidence of *Toxoplasma gondii* in Neural and Cardiac Tissues of Wild Rodents in Lithuania

**DOI:** 10.3390/pathogens14121252

**Published:** 2025-12-07

**Authors:** Giedrius Šidlauskas, Naglis Gudiškis, Dovilė Laisvūnė Bagdonaitė, Eglė Rudaitytė-Lukošienė, Evelina Juozaitytė-Ngugu, Marius Jasiulionis, Linas Balčiauskas, Dalius Butkauskas, Petras Prakas

**Affiliations:** State Scientific Research Institute Nature Research Centre, Akademijos 2, 08412 Vilnius, Lithuania; giedrius.sidlauskas@gamtc.lt (G.Š.); naglis.gudiskis@gamtc.lt (N.G.); dovile.bagdonaite@gamtc.lt (D.L.B.); egle.rudaityte@gmail.com (E.R.-L.); evelina.ngugu@gamtc.lt (E.J.-N.); marius.jasiulionis@gamtc.lt (M.J.); linas.balciauskas@gamtc.lt (L.B.); dalius.butkauskas@gamtc.lt (D.B.)

**Keywords:** *Toxoplasma gondii*, Lithuania, intermediate host, *ITS1* diversity, prevalence, molecular epidemiology

## Abstract

*Toxoplasma gondii*, a widespread parasite, poses significant public health concerns. It infects humans and animals, with rodents serving as important intermediate hosts. The present study investigated the prevalence and genetic *ITS1* diversity of *T*. *gondii* in wild rodents from Lithuania. A total of 469 rodents from eight species were captured across various regions, and DNA from neural and cardiac tissues was analyzed using nested PCR. Overall prevalence of *T*. *gondii* was 26.2% (95% CI = 22.3–30.5). The prevalence of infection varied among rodent species (0–50.0%) and across geographic locations. A mere few rodents exhibited concurrent infections in both tissues examined. *Toxoplasma gondii* was detected more frequently in the brains of *Apodemus flavicollis* and hearts of *Clethrionomys glareolus*, and in the males of *Microtus arvalis*. A total of 19 distinct *ITS1* genotypes were identified, including 17 novel ones; Genotype 1 was the most prevalent and widely distributed. Phylogenetic and network analyses revealed a star-like topology centered on Genotype 1 and confirmed the accurate identification of *T. gondii* in Lithuanian rodents. This study provides the first evidence of *T. gondii* in wild rodents in Lithuania, highlighting the need for further research on its prevalence and potential impact on public health and wildlife.

## 1. Introduction

*Toxoplasma gondii* is an obligate intracellular protozoan parasite that infects a wide range of warm-blooded animals, including humans [1]. Its life cycle involves both definitive hosts (DHs) and intermediate hosts (IHs), with members of the family Felidae serving as the DHs. In this host, the parasite undergoes sexual replication and sheds oocysts into the environment [2,3]. Excreted oocysts have the potential to contaminate soil, water, and food, thereby posing a considerable risk to IHs, including rodents, birds, and humans. The transmission of *T*. *gondii* occurs primarily through the ingestion of oocysts from contaminated sources, the consumption of tissue cysts in undercooked meat from infected animals, or transplacental transmission of tachyzoites from mother to offspring (congenitally) [4,5,6]. In addition, some studies have suggested that *T. gondii* may also be transmitted through the sexual route. Under natural conditions, transmission from males to females has been observed, although further research is required to confirm this mode of transmission [7]. In humans, *T*. *gondii* infection is frequently asymptomatic but can result in severe neurological or ocular diseases, particularly in immunocompromised individuals or during congenital transmission [8,9,10,11]. Given its widespread prevalence and potential health implications, *T*. *gondii* constitutes a major public health concern on a global scale.

Rodents, serving as IHs of *T*. *gondii*, play a pivotal role in the parasite’s transmission cycle. These animals frequently serve as prey for higher trophic predators, including carnivores and raptors, thereby acting as significant carriers in the parasite’s life cycle [12,13,14]. Rodents are highly responsive to environmental contamination and therefore serve as reliable bioindicators of *T. gondii* prevalence within ecosystems [15,16]. Their role as potential reservoir hosts highlights the importance of monitoring wild rodent populations to better evaluate environmental contamination and assess associated public health risks.

Tissue cysts of *T*. *gondii* are most prevalent in the neural and muscular tissues, including the brain, eyes, and skeletal and cardiac muscles [1]. These tissues are of particular interest due to their significance in chronic infection and the risk of transmission through the consumption of infected animals. Therefore, understanding the prevalence of *T*. *gondii* in these tissues can offer valuable insights for evaluating the broader public health implications of rodent-borne transmission.

*Toxoplasma gondii* is considered one of the most serious food-borne zoonoses in the world; however, there are substantial knowledge gaps concerning its distribution in wild rodent populations from the Baltic States [13,17]. Indeed, there is no genetic research on *T*. *gondii* in Lithuania from any hosts, and the parasite has been extensively understudied in this region. Thus, the aim of the present study was to detect *T*. *gondii* DNA in neural and cardiac tissue samples from rodents in Lithuania.

## 2. Materials and Methods

### 2.1. Sample Collection 

**Figure 1:**
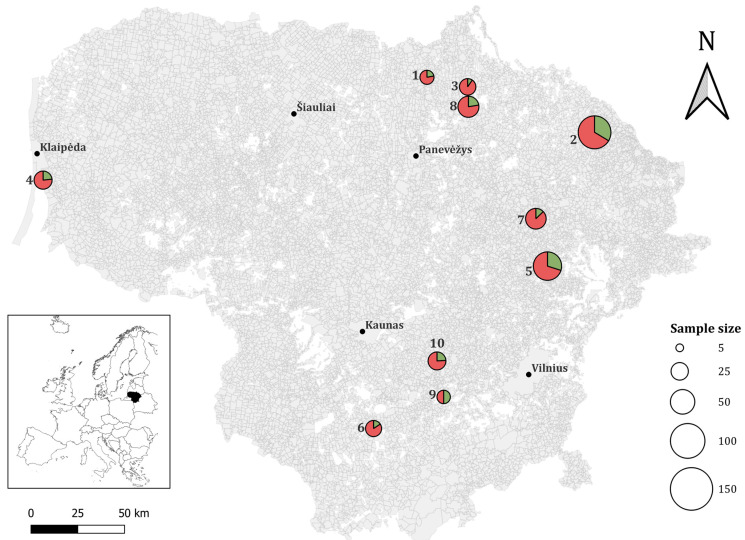
Geographic distribution of rodent sampling sites in Lithuania created with Quantum Geographic Information System (QGIS, “https://www.qgis.org (accessed on 15 July 2025)”): 1—Aukštikalniai, 2—Bileišiai, 3—Deikiškės, 4—Juodkrantė, 5—Kamasta, 6—Luksnėnai, 7—Lukštas, 8—Mieliūnai, 9—Zabarauskai, 10—Žiežmariai. Pie charts represent the prevalence of *T*. *gondii*: green segments indicate the proportion of positive samples, while red segments indicate the negative samples at each site.

In 2024, 469 wild rodents were captured for the investigation of *T*. *gondii* presence in rodent populations. Small mammals were trapped using snap traps, which is permitted for scientific research under Lithuanian law that result in instantaneous death, eliminating the need for subsequent euthanasia. Brown bread soaked in sunflower oil was used as bait in the traps. Exposition of traps was one–three days, each consisting of 25 traps spaced 5 m from each other. A total of five to ten trap lines were established at each study location. The trapping was part of multipurpose research, including the collection of meat and brain samples, reproductive parameters, and other biological materials. Most of the trapped small mammal species were identified based on their external features. *Microtus* and *Alexandromys* voles were distinguished by differences in their teeth [18] after cleaning their skulls. All *Microtus arvalis* were further referred to as *sensu lato*, since genetic methods were not employed to identify *Microtus rossiaemeridionalis* voles. Consequently, neural and cardiac tissues were not retrieved from all individuals, and in some cases, only one tissue type was successfully collected. All procedures were conducted under valid institutional and national ethical approvals. Ultimately, 401 brain samples and 401 heart samples were obtained from various wild rodent species across multiple locations in Lithuania, including Kamasta, Bileišiai, Juodkrantė, Žiežmariai, Deikiškės, Luksnėnai, Zabarauskai, Mieliūnai, Aukštikalniai, and Lukštas, for subsequent *T. gondii* screening. Detailed sample collection sites are indicated in Figure 1. 

Specimens represented eight rodent species across several genera: 45 striped field mouse (*Apodemus agrarius*), 167 yellow-necked mouse (*Apodemus flavicollis*), 1 tundra vole (*Alexandromys oeconomus*), 156 bank vole (*Clethrionomys glareolus*), 8 short-tailed field vole (*Microtus agrestis*), 73 common vole (*M*. *arvalis sensu lato*), 15 Eurasian harvest mouse (*Micromys minutus*), and 4 house mouse (*Mus musculus*). Rodents were trapped in natural meadows, forests, shrubby clearings, and orchards. Approval of this study was granted by the Animal Welfare Committee of the State Scientific Research Institute Nature Research Centre (Protocol No. GGT-9, dated 12 January 2024).

### 2.2. gDNA Extraction and Molecular Analysis

Neural tissues were subjected to gDNA extraction using the FastPure Blood/Cell/Tissue/Bacteria DNA Isolation Mini Kit (Vazyme, Nanjing, China), while cardiac samples were extracted using ThermoFisher Genomic DNA Purification Kit (ThermoFisher Scientific Baltics, Vilnius, Lithuania) in accordance with the manufacturer’s protocol for DNA extraction from tissue samples. DNA concentration and purity were assessed using a Nanophotometer P330 (Implen, Munich, Germany). *Toxoplasma gondii* was detected by amplifying the partial internal transcribed spacer 1 (*ITS1*) region using nested PCR (nPCR) with the primer pairs designed during this study. For the first round of nPCR, the external primer pair of TgNN3/TgNN5R was used, followed by a second round using the internal primer pair of TgNP3/TgNP5R to amplify the target fragment (Table 1). 

The first round of nPCR reaction was carried out in a 25 µL reaction volume, consisting of 12.5 µL Taq Master Mix (Vazyme, Red Maple Hi-tech Industry Park, Nanjing, China), 7.5 µL nuclease-free water, 0.5 µL of each external primer, with a final addition of 4 µL of the extracted DNA. The second step of the nPCR was also performed in a 25 µL reaction volume, with the nPCR mixture containing 12.5 µL Taq Master Mix (Vazyme, Red Maple Hi-tech Industry Park, Nanjing, China), 9.5 µL nuclease-free water, 0.5 µL of each internal primer. However, instead of extracted gDNA, 2 µL of the first PCR product was used. Water was used as the negative control instead of the template DNA for both steps of the nested PCR. The gDNA of *T*. *gondii* obtained from the culture media served as a positive control in this study.

Both rounds of the nPCR were conducted according to the manufacturer’s protocol: beginning with the initial denaturation step at 95 °C for 3 min, followed by 35 cycles consisting of 15 s of denaturation at 95 °C, 15 s of annealing at 60–61 °C (depending on the primer pair used), and 60 s of elongation at 72 °C. A final extension was performed at 72 °C for 5 min. The success of the reaction was tested using 1% agarose gel electrophoresis, and positive amplicons were purified using ExoI (Thermo Fisher Scientific Baltics, Vilnius, Lithuania) and FastAP (Thermo Fisher Scientific Baltics, Vilnius, Lithuania), following the manufacturer’s instructions.

### 2.3. Sequencing Analysis

All positive samples were subjected to direct sequencing using Big-Dye^®^ Terminator v3.1 Cycle Sequencing Kit (Thermo Fisher Scientific, Vilnius, Lithuania) and the 3500 Genetic Analyzer (Applied Biosystems, Foster City, CA, USA). Both forward and reverse primers of the second round of nPCR were employed for Sanger sequencing. All the acquired chromatograms were pure, without double peaks or polysignals. All sequences acquired in this study are available in the NCBI GenBank database under accession numbers PX571996–PX572122.

### 2.4. Bioinformatical and Statistical Analysis

To identify and compare acquired sequences with those of *T*. *gondii*, the Nucleotide BLAST tool (https://blast.ncbi.nlm.nih.gov/, accessed on 15 September 2025) was utilized. MEGA12 12.0.14 [19] software was used to align the *ITS1* sequences obtained in this study with those available in the NCBI GenBank database, employing the MUSCLE algorithm for the alignment. The determination of best fitting model and construction of the Bayesian phylogenetic tree were executed using TOPALi v2.5 software [20]. The phylogenetic tree was constructed using a Kimura 80 model. The final alignment of *ITS1* sequences included 58 individual sequences and resulted in 268 aligned nucleotide positions including gaps. The phylogenetic network analysis of *ITS1* genotypes was conducted using the median joining method [21] implemented in NETWORK 10.2.0.0 software (https://www.fluxus-engineering.com/sharenet.htm, accessed on 7 October 2025). 

The indices of intraspecific genetic variability, the number of genotypes (*h*), the number of segregating/polymorphic sites (*S*), the average number of nucleotide differences (*K*), the haplotype diversity (*Hd*), the nucleotide diversity (*π*), and the standard deviation (*SD*) for the last two parameters, as well as values of Tajima’s D neutrality test [22] were calculated with DnaSP v. 6. 12. 03 software [23]. Genetic differentiation based on pairwise distances was evaluated using *F_ST_* with Arlequin v. 3.5.2.2 [24]. The statistical significance of each pairwise *F_ST_* was tested by 10,000 permutations at the 95% confidence level.

We calculated the prevalence estimates and corresponding 95% confidence intervals (CIs) for *T*. *gondii* infection across various categories, including species, sample collection sites, sex, and age groups. Statistical comparisons were performed using the online G-test (https://elem.com/~btilly/effective-ab-testing/g-test-calculator.html, accessed on 15 July 2025). Overall comparisons included data from all samples; however, comparative analysis focused only on species that were represented by more than 40 individuals. Additionally, genotype-specific prevalence and 95% CIs were calculated based on sample collection sites and the different rodent species investigated. Statistically significant differences were assessed using an online Fisher’s exact two-tailed test (https://www.socscistatistics.com/tests/fisher/default2.aspx, accessed on 15 July 2025), with significance level defined as *p* < 0.05.

## 3. Results

### 3.1. Prevalence of T. gondii in Wild Rodent Species in Lithuania

In this study, 469 individuals representing eight different species (*A*. *agrarius*, *A*. *flavicollis*, *A*. *oeconomus*, *C*. *glareolus*, *M*. *agrestis*, *M*. *arvalis*, *M*. *minutus*, and *M*. *musculus*) were analyzed for *T*. *gondii* infection. Molecular analysis detected at least one positive specimen for *T*. *gondii* DNA in six of the species examined. However, the sample size for two species (*M*. *musculus* and *A*. *oeconomus*) that were negative for *T*. *gondii* was below five individuals, making them underrepresented in this study (Table S1).

Out of 469 individuals studied, 26.2% (123/469; 95% CI = 22.3–30.5) were positive for *T*. *gondii* infection (Table 2 and Table S1). Among those, only 0.9% (4/469; 95% CI = 0.2–2.2) had both of their organs infected. When considering both neural and cardiac tissues, the overall prevalence was 15.8% (127/802; 95% CI = 13.4–18.6), accounting for the inclusion of paired organ samples per individual. *Toxoplasma gondii* was determined in the northern, southern, western, and eastern parts of Lithuania (Figure 1). The highest detection rates were established in Zabarauskai (southern part of Lithuania) with 50.0% (95% CI = 11.8–88.2), in Bileišiai (eastern part of Lithuania) with 33.6% (95% CI = 25.9–41.4) and in Kamasta (eastern part of Lithuania) with 29.9% (95% CI = 21.2–39.8). 

Among the six species positive for *T*. *gondii*, infection rates varied from 17.8% to 50.0%. However, differences in prevalence among host species were not statistically significant (G = 2.88, *p* > 0.05). The result remained non-significant even after excluding the two less-represented species, *M. agrestis* and *M. minutus* (G = 1.59, *p* > 0.05). No statistically meaningful differences in *T*. *gondii* detection rates were observed when comparing overall prevalence in cardiac (17.0%) and neural tissues (14.7%) (G = 0.59, *p* > 0.05). Further analysis revealed no discernible difference in *T*. *gondii* prevalence between cardiac and neural tissues in *A*. *agrarius* (G = 0.44, *p* > 0.05) and *M. arvalis* (G = 0.34, *p* > 0.05). Meanwhile, *T*. *gondii* was more frequently detected in the brains (19.5%) than in the hearts (8.7%) of *A. flavicollis* (G = 6.02, *p* < 0.02). In contrast, *T*. *gondii* was more commonly found in the hearts (23.3%) than in the brains (11.2%) of *C. glareolus* (G = 5.99, *p* < 0.02).

Comparison of *T. gondii* prevalence rates in cardiac tissues between each positive species revealed notable statistical differences (G = 15.94, *p* < 0.008). Even after exclusion of two species (*M. agrestis*, *M. minutus*) that were represented by less than 20 individuals, statistically significant differences remained (G = 10.89, *p* < 0.02). Comparative analysis revealed that *C. glareolus* cardiac samples were more likely to test positive for *T. gondii* than *A. flavicollis* samples (G = 9.35, *p* < 0.003). Meanwhile, analysis of neural tissues revealed that all four species positive for *T*. *gondii* had similar likelihood to be infected (G = 3.29, *p* > 0.05). Pairwise comparative analysis between positive species did not reveal any statistically significant differences.

The overall prevalence did not differ significantly among juveniles (26.1%), sub-adults (28.3%), and adults (25.4%) (G = 0.22, *p* > 0.05). Further comparison of age groups among the four positive and well represented species revealed no statistically significant differences in parasite detection among juvenile (20.1–28.0%) (G = 0.13, *p* > 0.05), subadult (12.5–37.5%) (G = 2.24, *p* > 0.05) or adult (20.0–34.0%) (G = 2.47, *p* > 0.05) specimens. Similarly, no significant differences in prevalence among age groups were observed within *A*. *agrarius* (G = 0.22, *p* > 0.05), *A. flavicollis* (G = 1.26, *p* > 0.05)*, C. glareolus* (G = 0.80, *p* > 0.05) or *M. arvalis* (G = 0.96, *p* > 0.05). A further comparative analysis, using the pairwise method, confirmed the initial findings, as no significant differences in age groups within and between the species were identified.

No statistically significant differences in overall prevalence were observed between male (22.8%) and female (22.6%) specimens (G = 1.93, *p* > 0.05). Further comparison of the four best-represented species revealed no significant differences in parasite detection between male (G = 4.60, *p* > 0.05) or female (G = 4.31, *p* > 0.05) samples. No significant sex-related differences in *T*. *gondii* prevalence were observed in *A*. *agrarius* (G = 0.22, *p* > 0.05), *A. flavicollis* (G = 2.38, *p* > 0.05) and *C. glareolus* (G = 0.85, *p* > 0.05). In contrast, analysis of *M. arvalis* samples indicated that males (48.2%) were significantly more likely to test positive than females (16.3%) (G = 6.67, *p* < 0.01).

### 3.2. Distribution of ITS1 Genotypes

In total, 19 distinct *T*. *gondii ITS1* genotypes were identified across the samples analyzed (Table 3). Among these, 13 genotypes were detected in cardiac tissues and eight in neural tissues. Notably, only two *ITS1* genotypes (Genotype 1 and Genotype 35) were shared between both tissue types. Genotype 1 was the most prevalent, being detected in 84.3% (107/127; 95% CI = 76.7–90.1) of all *T. gondii*-positive samples. There was no statistically significant difference in Genotype 1 prevalence between cardiac and neural tissues (Fisher’s exact test two-tailed, *p* > 0.05). The greatest genotype diversity in cardiac samples was detected in Bileišiai, where six distinct genotypes were identified (Genotypes 1, 38, 39, 41, 42, and 49), followed closely by Kamasta with five genotypes (Genotypes 1, 30, 34, 40 and 50). Similarly, neural samples from Kamasta exhibited the highest genotype variability, comprising four genotypes (Genotypes 1, 35, 44, and 46), while Bileišiai neural samples contained three distinct genotypes (Genotypes 1, 35, and 47). Samples from *A. flavicollis* and *C. glareolus* exhibited the highest *ITS1* genotype variability, with five genotypes each in cardiac and neural tissues for *A. flavicollis*, and six in cardiac and three in neural tissues for *C. glareolus*. However, no statistically significant differences in genotype composition were observed in this study (Fisher’s exact two-tailed test, *p* > 0.05).

Interestingly, concurrent infection of both cardiac and neural tissues was observed in only four investigated individuals: one *C*. *glareolus*, one *A. agrarius*, and two *M*. *arvalis* specimens. However, the genotype composition varied between individuals. In one *M. arvalis* and one *A*. *agrarius* specimen, both tissues were infected with *T*. *gondii ITS1* Genotype 1. In the second *M. arvalis*, cardiac tissue was infected with Genotype 42, while neural tissue harbored Genotype 1. The *C. glareolus* individual showed a mixed infection pattern as well, with Genotype 49 detected in the cardiac tissue and Genotype 1 in the neural tissue.

### 3.3. Genetic Variability of T. gondii from Rodents in Lithuania

**Figure 2:**
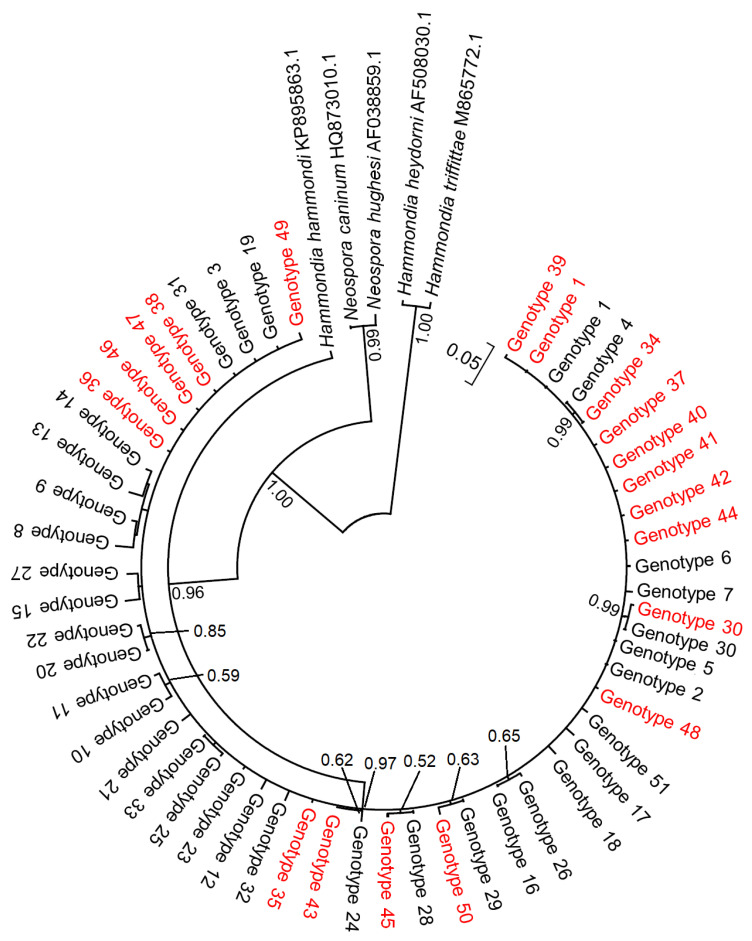
Circular phylogenetic tree of selected species from the family Sarcocystidae based on partial *ITS1* sequences. The tree was constructed using the Bayesian method, rooted with *Hammondia heydorni* and *H. triffittae*, and scaled according to branch length. The Kimura 80 evolutionary model was applied. In total, 51 *ITS1* genotypes of *Toxoplasma gondii* were included, and the sequences generated in the present study are shown in red. Posterior probability values greater than 0.5 are displayed next to the branches.

Based on the comparison of the 239 bp *ITS1* sequences generated in this study, 19 genotypes were identified, of which only Genotype 1 and Genotype 30 showed 100% identity with *T. gondii* sequences reported from other countries. Thus, 17 genotypes were detected for the first time in the current study. After analyzing all *ITS1* sequences available in the GenBank database, we identified 51 genotypes (Table S2). The alignment of the following genotypes revealed 63 polymorphic sites, 3 of which had deletions in one of the sequences (Table S3). According to BLAST results, our sequences displayed 96.2–100% similarity to previously published *T*. *gondii* sequences. The Bayesian phylogenetic tree generated using *ITS1* sequences confirmed the accurate identification of *T*. *gondi* in analyzed animals from Lithuania (Figure 2). The grouping of 51 *T*. *gondii* genotypes was supported by 0.97 posterior probability value. Based on the partial *ITS1* region examined, *T*. *gondii* clustered with *Hammondia hammondi* with a high support (0.96). No clear structuring was observed among the analyzed *T. gondii ITS1* genotypes, except that Genotypes 4 and 34, as well as Genotypes 20 and 22, formed robust clusters.

**Figure 3:**
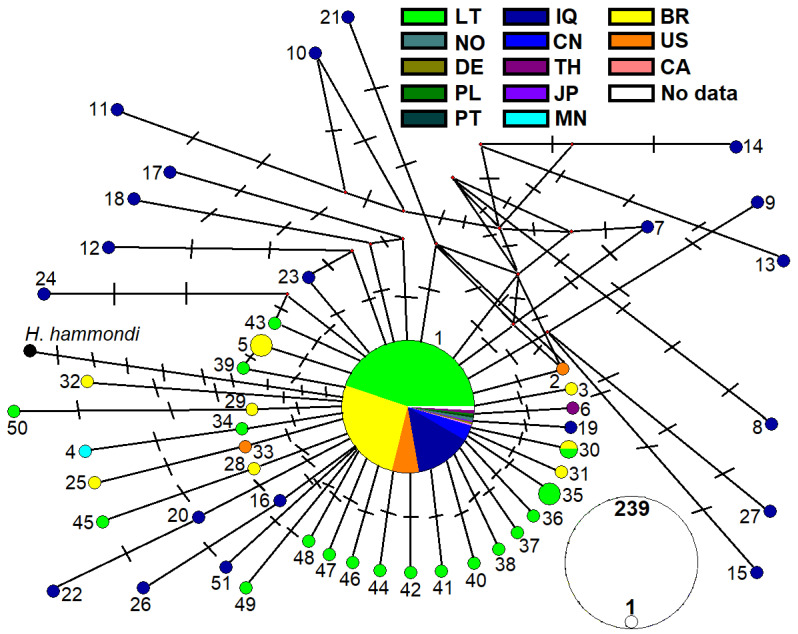
Median joining network of *T*. *gondii ITS1* genotypes. The size of each circle is proportional to the genotype frequency, and dashes represent individual mutational steps. Red circles denote hypothetical genotypes representing unsampled or extinct ancestral haplotypes inferred in the network. The different colours represent isolation countries: LT—Lithuania, NO—Norway, DE—Germany, PL—Poland, PT—Portugal, IQ—Iraq, CN—China, TH—Thailand, JP—Japan, MN—Mongolia, BR—Brazil, US—USA, CA—Canada.

The network displays a predominantly star-like topology with a single large central node (Genotype 1) connected to many peripheral genotypes through one to several (two to six) mutational steps (Figure 3). Genotype 1 was linked by a single mutational step to slightly more than half of the remaining genotypes (26 in total). The central Genotype 1 was detected in samples from nearly all countries, except Mongolia. The overall frequency of other genotypes reached only 18.7%. In addition to Genotype 1, several other genotypes (Genotypes 5, 30, and 35) were represented by more than one sequence.

Geographic structuring within the network is weak but still discernible. Isolates originating from Asia (China, Japan, Thailand, and Mongolia) clustered toward the central part of the network, indicating limited regional divergence. Brazilian and North American genotypes (from the USA and Canada) either corresponded to the dominant Genotype 1 or were positioned close to the central node, differing from Genotype 1 by one to three mutational steps. The examined European isolates from Norway, Germany, Portugal, and Poland were assigned exclusively to Genotype 1. In contrast, the most divergent genotypes were those detected in Iraq were separated from the central node by several mutational steps and up to 12 hypothetical intermediate genotypes.

Lithuanian isolates are particularly abundant and occupy both central and peripheral positions in the network. Three Lithuanian genotypes (Genotypes 45, 49, and 50) differed from the dominant genotype by two–three mutational steps. Interestingly, Genotypes 39, 45, and 50 show a close relationship to Brazilian genotypes, while Genotype 34 is closely related to Genotype 4, which was detected in Mongolia. Furthermore, besides the dominant genotype, Genotype 30 was found in isolates from more than one country, Brazil and Lithuania.

The analysis of partial *ITS1* sequences of *T*. *gondii* revealed moderate genetic variability (Table 4). The calculated values of overall haplotype and nucleotide diversity (*Hd* = 0.313 ± 0.036; *π* = 0.00268 ± 0.00042) indicate the presence of limited but detectable genetic variation across all examined samples. The highest genetic variation was found in sample from Iraq (*K* = 1.88060, *Hd* = 0.631, *π* = 0.00956), while other regional samples displayed seemingly lower variation. In the Lithuanian dataset, overall haplotype diversity (*Hd* = 0.291 ± 0.034) and nucleotide diversity (π = 0.00158 ± 0.00036) were slightly higher than in Brazil and the USA, whereas no variation was detected among nine sequences from China. Within the Lithuanian dataset, the greatest diversity was found in *A*. *flavicollis* heart samples (*Hd* = 0.618 ± 0.164, π = 0.00456 ± 0.00187).

Most datasets showed negative Tajima’s D values, with significantly negative values detected for the overall dataset as well as for Europe, Asia, the Americas, Brazil, Iraq, and Lithuania. Within Lithuania, negative Tajima’s D values were also observed, with statistical significance in *A*. *flavicollis* and *C*. *glareolus* heart samples.

The calculated pairwise *F_ST_* values were insignificant for all compared samples, except for the comparison between *A*. *flavicollis* heart and brain samples collected in Lithuania (*F_ST_* = 0.056, *p* < 0.05). This finding indicates moderate genetic differentiation between these two datasets.

## 4. Discussion

### 4.1. Trends in the Prevalence of Toxoplasma gondii in Wild Rodents in Europe

During this study, various species of wild rodents from Lithuania were tested for the presence of *T*. *gondii* infection. The overall prevalence of *T*. *gondii* among the examined rodents was 26.2% as determined by molecular analysis targeting *ITS1* gene. Although there are currently no studies investigating the same group of wild rodent species analyzed in the present study, the observed prevalence aligns with the general *T. gondii* prevalence patterns reported in other wild rodent species, which range from 0% to 83.3% [13]. Such a wide variation in prevalence is largely attributable to substantial differences in sample sizes across studies, varying from as few as 10 individuals to more than 1000. Consequently, direct comparison of the present results with other investigations worldwide is constrained by methodological differences, including the choice of sampled tissues and genetic markers.

Globally, molecular studies investigating *T*. *gondii* infection patterns in wild rodents remain limited compared to the numerous serological surveys available. In Europe, molecular data on *T. gondii* infection in rodents are available from Austria, the Czech Republic, Germany, Romania, Slovakia, Slovenia, Switzerland, and Russia [25,26,27,28,29,30,31,32]. The reported prevalence of *T*. *gondii* in *A. agrarius* populations ranges from 0% to 12.5% (95% CI = 0.65–53.3), whereas *A. flavicollis* shows lower infection rates, reaching up to 9.1% (95% CI = 0.47–42.9) [29,30,31,32,33,34,35]. In the present study, *A. flavicollis* exhibited a higher prevalence (25.2% (95% CI = 18.8–32.4)) than *A. agrarius* (17.8% (95% CI = 8.0–32.1)), both markedly exceeding the infection rates reported in previous European studies. The overall prevalence of *T*. *gondii* in *C. glareolus* in our study was 28.2% (95% CI = 21.3–36.0), which is comparatively high relative to earlier reports indicating infection rates between 0% and 13.9% (95% CI = 10.9–17.5) [31,36,37]. The finding that 50% (95% CI = 15.7–84.3) of the examined *M. agrestis* individuals were positive for *T. gondii* aligns with results from Germany [35]. The prevalence rate observed in *M. arvalis* (27.4% (95% CI = 17.6–39.1)) also corresponds to data from Germany (27.3% (95% CI = 9.7–56.6)) [35], while previous European studies have reported lower infection rates ranging from 0% to 4.2% (95% CI = 0.7–15.3) [25,36,38]. In the Netherlands, only one *M. arvalis* specimen was examined, which tested positive for *T. gondii* [33]. Furthermore, one *M. agrestis* and four *M. arvalis* specimens were examined in Croatia, but *T. gondii* infection was not detected in any of them [37]. Regarding *M. minutus*, only one European study to date has investigated the prevalence of *T. gondii* infection, reporting a rate of 11.1% (95% CI = 0.3–48.3) in Romania [32]. In contrast, the present study revealed a considerably higher prevalence of 33.3% (95% CI = 11.8–61.6) in *M. minutus* from Lithuania. Additionally, we also tested four individuals of *M*. *musculus* and one specimen of *A*. *oeconomus*; however, all were negative, likely due to the small sample size.

In this study, no statistically significant variation in prevalence was observed among the overall age groups, sexes, or examined tissues of the samples, nor between host species (Table 2 and Table S1). However, certain species-specific patterns were observed. The prevalence of *T. gondii* infection was significantly higher in males of *M. arvalis* than in females, suggesting that sex may influence exposure or susceptibility. Male-biased infection likely reflects behavioral ecology, as males typically have larger ranges and greater mobility, especially during breeding [39], increasing contact with contaminated soil or vegetation, though this pattern is not universal [40]. Additionally, testosterone-mediated immunosuppression may enhance susceptibility to infection [41]. Together, these results support the hypothesis that ecological exposure and hormonal factors influence *T. gondii* prevalence patterns in *M. arvalis* populations. Sex-related differences in *T. gondii* infection have been reported across various rodent species, though patterns are inconsistent. In Polish *C. glareolus* populations, females showed higher seroprevalence than males suggesting hormonal or behavioral modulation of infection risk [42]. Conversely, in Slovakian study, *M. arvalis* and other species showed the opposite pattern, where females exhibited significantly higher seropositivity compared to males [41]. Additionally, female guinea pigs (*Cavia porcellus*) in Colombia were significantly more likely to be positive [43], whereas in Armenia, 18.4% of male rodents were infected, with no infections detected in females [44]. Other studies found no significant sex differences [25,32,45], suggesting that these patterns vary among populations.

### 4.2. Ecological and Tissue-Specific Dynamics of Toxoplasma gondii in Wild Rodents

Geographically, higher parasite infection rates were detected in eastern sampled regions of Lithuania compared to western, northern, and southern sampling sites (Figure 1). Considering the geographical distribution of *T*. *gondii*, the highest detection rate was observed in Zabarauskai (50%; 3/6; 95% CI = 11.8–88.2), while the lowest rates were recorded in Deikiškės (9.5%; 2/21; 95% CI = 1.18–30.4) and Lukštas (10.9%; 5/46; 95% CI = 3.6–23.6). However, due to significant differences in sample sizes across the locations, a direct comparison of the detection rates is not feasible. Rodents function as both IHs and potential sources of infection for domestic cats and the Eurasian lynx (*Lynx lynx*), the only wild feline predator in Lithuania [46]. The detection of *T*. *gondii*-positive rodents in several locations signifies their epidemiological importance. Environmental data (biotope, farmstead proximity) and felid presence (based on motion-activated camera traps, animal tracks, and known lynx habitats) were obtained for all *T*. *gondii*-positive individuals (Table S4). Lynxes were observed in habitats corresponding to 84 *T*. *gondii*-positive individuals, whereas domestic cats were recorded in areas associated with 10 positive individuals. No signs of felid presence were detected in sites where 29 *T*. *gondii*-positive individuals were captured. Although the rodent species investigated in this study are characterized by small home ranges and limited dispersal abilities, lynxes are mobile predators that actively approach their prey, particularly when prey availability is low [47]. Additionally, the use of domestic predators (e.g., cats) to control rodent populations around farms and homesteads is a widespread practice globally [48]. However, domestic cats often exhibit limited predation on rodents, as regular human-provided food reduces their hunting motivation and dependence on wild prey [49]. Thus, further ecological and molecular investigations are required to provide a more comprehensive understanding of *T. gondii* transmission dynamics among wild rodents in Lithuania, particularly targeting lynxes, which may play a crucial role in the transmission cycle.

Among the 469 rodents examined, *T*. *gondii* DNA was detected in 123 individuals, of which only four showed concurrent infection in both brain and heart tissues. No statistically significant difference in *T*. *gondii* detection rates was observed between brain and heart samples overall, nor within *A*. *agrarius* and *M*. *arvalis* species (Table 2). However, some species-specific patterns were evident: *T*. *gondii* was more frequently detected in the brains of *A*. *flavicollis* than in their hearts, and vice versa—heart infections were more prevalent in *C*. *glareolus* than in their brains. These contrasting patterns suggest that host biology may influence tissue-specific parasite distribution, potentially reflecting differences in immune regulation, tissue physiology, or behavioral exposure pathways. Additionally, infection dose and transmission route may influence the initial site of dissemination and subsequent tissue affinity. For instance, congenital transmission often results in central nervous system involvement, whereas oral ingestion of oocysts or tissue cysts typically leads to systemic hematogenous spread [50,51]. Dual-organ infections were rare, detected in only four individuals, which may indicate a strong tissue preference or a sequential infection dynamic. Comparable findings were reported in Benin, where *T*. *gondii* DNA was detected in 15.2% of 632 small mammals, yet only 13 animals (13.5% of positives) were infected in both brain and heart tissues [52]. Such evidence highlights the value of examining both organs concurrently in future small-mammal surveys to obtain more accurate estimates of parasite prevalence. Another study examining two rodent species in Tunisia also demonstrated that the prevalence of *T*. *gondii* DNA varied among tissues, with the highest detection in the brain (21.4%; 3/14; 95% CI = 4.7–50.8), followed by muscle (18%; 8/43; 95% CI = 8.4–33.4), heart (16%; 7/43; 95% CI = 6.8–30.7), and spleen (11%; 5/43; 95% CI = 3.9–25.1) [53]. Infections were frequently confined to a single tissue, although some rodents exhibited multi-organ involvement, with up to four tissues testing positive in the same individual. In the present study, two individuals in whom *T*. *gondii* was detected in both organs exhibited distinct *ITS1* genotypes in different tissues, suggesting possible superinfection, where hosts acquire *T*. *gondii* through repeated exposure [54,55]. Similar mixed-genotype infections have been reported in wildlife and domestic hosts, such as feral cats and pigs, reflecting a broader pattern of multi-strain circulation [56,57]. In enzootic environments, repeated exposure to genetically diverse strains promotes genetic diversity and facilitates sexual recombination. Theoretical modeling also suggests that repeated exposure to oocysts from diverse felid sources can lead to persistent co-infections, which may influence strain dynamics at the population level [58]. Experimental models in chronically infected mice further demonstrate how virulent strains can evade immunity [55]. Therefore, our findings of genetically distinct *T*. *gondii* DNA within the same host are consistent with observed patterns of co-infections in natural systems, highlighting the complexity of *T*. *gondii* transmission in Lithuanian rodent populations.

### 4.3. Genetic Diversity and Ecological Implications of Toxoplasma gondii in Lithuanian Rodents

In Lithuania, *ITS1* Genotype 1 of *T*. *gondii* was the predominant variant in neural and cardiac tissues of wild rodents, being detected in 85.6% of all *T*. *gondii*-positive samples. The remaining 18 *ITS1* genotypes differed from the central dominated genotype by one to three mutations. Based on the comparison of *ITS1* sequences of *T*. *gondii* available in GenBank, a predominantly clonal population structure was evident, with the cosmopolitan Genotype 1 detected in 81.3% of all samples (Figure 3). Numerous rare, mostly singleton haplotypes branched out from this central genotype, showing a star-like network. This pattern supports earlier findings of clonal dominance in *T*. *gondii* and is consistent with models implicating limited recombination and selection in its global distribution [59]. The generally low nucleotide diversity and significantly negative Tajima’s D values across regions, including Lithuania (Table 4), reinforce a scenario of demographic expansion or purifying selection acting on *ITS1*. Comparable negative Tajima’s D values have also been reported from the *B1* and *ROP8* genes of *T*. *gondii* [60]. Thus, the global dominance of *ITS1* Genotype 1 may reflect evolutionary mechanisms that favour its persistence, including purifying selection or historical demographic events leading to genetic drift-induced fixation. However, no studies to date have attempted to deepen our understanding by exploring the evolutionary mechanisms underlying the dominance of Genotype 1. Therefore, future research that combines genetic and experimental approaches would be crucial to clarify why Genotype 1 remains so widespread across diverse regions and hosts, and whether its prevalence is linked to functional traits that influence pathogenicity and transmission.

In our investigation, all *T*. *gondii*-positive rodents were trapped within one and a half kilometers of residential dwellings (Table S4), and 26.2% (95% CI = 22.3–30.5) of the tested individuals were found to be infected (Table 2). Due to the *T*. *gondii* bradyzoite’s ability to persist throughout the lifetime of the infected host, rodents are a key in sustaining the parasite’s circulation across domestic and wild transmission cycles [61]. While rodents themselves rarely constitute a direct risk to humans, their role as prey for cats or lynxes may indirectly enhance environmental contamination with *T*. *gondii* oocysts, thereby increasing the potential for human exposure [62]. The moderate prevalence observed in our study indicates that *T*. *gondii* could be maintained through a rodent–cat–human transmission route, emphasizing the possible contribution of peri-domestic rodent populations to the spread of toxoplasmosis. However, comparing our results with those of other studies requires caution, as reported prevalence rates in wild rodents often vary according to the diagnostic methods, sample size, and molecular markers used. Such methodological variability can substantially influence detection sensitivity and, consequently, the apparent infection rates reported across regions and species. Current studies have documented *T*. *gondii* prevalence in a range of wild rodent species; yet these investigations are often constrained by the analytical techniques applied. Molecular approaches used to assess *T*. *gondii* prevalence rely on different genetic markers, including the *B1*, *GRA5*, *TGR1E*, and *18S* rRNA genes, as well as the *ITS1* region and the 529 bp repetitive element in the *T*. *gondii* genome [26,29,37,44,63]. Various modifications of PCR are employed globally, such as conventional PCR, RT-PCR, and qPCR [31,35,55], each differing in sensitivity and specificity. Furthermore, a greater proportion of *T*. *gondii* prevalence data originates from serological investigations, which have been extensively carried out across diverse geographical regions and host species [41,64,65]. These studies have considerably expanded the global understanding of infection dynamics in IHs. Nevertheless, serological assays were not included in the present work, and therefore our results pertain exclusively to molecular detection. In this context, it should also be noted that the present study did not assess the infectivity of the detected stages, which limits inferences regarding parasite viability. Indeed, not all *T*. *gondii* DNA detections can be definitively correlated with the presence of viable tissue cysts. However, positive PCR results targeting the *ITS1* gene provide strong evidence of *T*. *gondii* stages, supporting the reliability of molecular detection in our analysis. Additionally, we attempted to genotype the *T*. *gondii*-positive samples targeting the *GRA5*, *PK1*, and *L358* genetic markers. Nevertheless, no successful genotyping results were obtained. This outcome may be related to the low DNA concentrations in the samples, which likely hindered the amplification of genotyping fragments. Future studies should incorporate viability assays to better determine whether detected stages represent infectious parasites. Expanded multilocal genotyping will also help to clarify the diversity and circulation of *T*. *gondii* in wildlife reservoirs.

To conclude, our study demonstrates that six of the eight wild rodent species examined in Lithuania are susceptible to *T*. *gondii* infection. Molecular analysis targeting *ITS1* gene revealed several novel *T*. *gondii* genotypes, thereby expanding the current database of nucleotide sequences and contributing to a broader understanding of the parasite’s genetic diversity.

## Figures and Tables

**Table 1:** Primers used in the present study to amplify the *ITS1* region fragments of *T. gondii*.

Primer Name	Sequence 5′-3′	Region	Fragment Size	nPCR Round
TgNN3	GAATCCCAAGCAAAACATGAG	*ITS1*	384 bp	I
TgNN5R	CCAAGACATCCATTGCTGAAA	*5.8S* rRNA		
TgNP3	AAGCGTGATAGTATCGAAAGG	*ITS1*	281 bp	II
TgNP5R	GAAGCAATCTGAAAGCACATC	*ITS1*		

**Table 2:** Prevalence rates of *T*. *gondii* infection in investigated rodent samples.

		Species								Overall
		*A. agrarius*	*A*. *flavicollis*	*C. glareolus*	*M. arvalis*	*M. agrestis*	*M. minutus*	*M. musculus*	*A. oeconomus*	*n*/*N* (%, CI)
**Tissue**	Heart	9.1 (2.5–21.7)	8.7 (4.4–15.0)	23.3 (16.3–31.5)	19.2 (11.0–30.1)	50.0 (15.7–84.3)	33.3 (11.8–61.6)	0	0	68/401 (17.0, 13.4–21.0)
	Brain	17.2 (5.9–35.8)	19.5 (13.7–26.5)	11.2 (6.4–17.8)	13.8 (6.2–25.4)	0	0	0	0	59/401 (14.7, 11.4–18.6)
**Age**	Juvenile	27.3 (6.0–61.0)	28.0 (12.1–49.4)	27.3 (16.1–41.0)	20.1 (7.2–42.2)	*N/A*	*N/A*	*N/A*	*N/A*	30/115 (26.1, 18.3–35.1)
	Sub-adult	12.5 (1.6–38.4)	30.6 (18.3–45.4)	23.9 (12.6–38.8)	37.5 (18.8–59.4)	60.0 (14.7–94.7)	*N/A*	*N/A*	*N/A*	39/138 (28.3, 20.9–36.6)
	Adult	20.0 (4.3–48.1)	20.7 (12.9–30.4)	34.0 (21.5–48.3)	25.0 (9.8–46.7)	33.3 (0.8–90.6)	66.7 (9.4–99.2)	0	0	50/197 (25.4, 19.5–32.1)
	Undet.	0	100 (2.5–100)	0	0	*N/A*	25.0 (5.5–57.2)	*N/A*	*N/A*	4/19 (21.1, 6.1–45.6)
**Sex**	Female	13.6 (2.1–34.9)	18.1 (10.0–28.9)	32.2 (20.6–45.6)	16.3 (6.8–30.7)	60.0 (14.7–94.7)	50.0 (1.3–98.7)	0	*N/A*	46/204 (22.6, 17.0–28.9)
	Male	23.8 (8.2–47.2)	29.7 (20.6–40.2)	23.9 (15.4–34.1)	48.2 (28.7–68.1)	33.3 (0.8–90.6)	50.0 (1.3–98.7)	0	0	68/236 (22.8, 23.1–35.1)
	Undet.	0	50.0 (6.8–93.2)	44.4 (13.7–78.8)	0	*N/A*	27.3 (6.0–61.0)	*N/A*	*N/A*	7/29 (24.1, 10.3–43.54)
**Overall**		8/45 (17.8, 8.0–32.1)	42/167 (25.2, 18.8–32.4)	44/156 (28.2, 21.3–36.0)	20/73 (27.4, 17.6–39.1)	4/8 (50.0, 15.7–84.3)	5/15 (33.3, 11.8–61.6)	0/4	0/1	123/469 (26.2, 22.3–30.5)

Undet.—undetermined, *N/A*—not applicable, *n*—number of *T*. *gondii*-positive samples, *N*—number of total samples investigated.

**Table 3:** Frequency of *Toxoplasma gondii ITS1* genotypes identified in cardiac and neural tissues of various rodent species.

Genotype Type	Prevalence Rate of Genotype Type in Cardiac Tissue (95% CI)	Prevalence Rate of Genotype Type in Neural Tissue (95% CI)
Genotype 1	*Apodemus agrarius* 6.8% (95% CI = 1.4–18.7) *Apodemus flavicollis* 5.5% (95% CI = 2.2–11.0) *Clethrionomys glareolus* 19.4% (95% CI = 13.0–27.3) *Microtus agrestis* 50.0% (95% CI = 15.7–84.3) *Microtus arvalis* 16.4% (95% CI = 8.8–27.0) *Micromys minutus* 33.3% (95% CI = 11.8–61.6)	*Apodemus agrarius* 13.8% (95% CI = 3.9–31.7) *Apodemus flavicollis* 17.0% (95% CI = 11.5–23.7) *Clethrionomys glareolus* 9.7% (95% CI = 5.3–16.0) *Microtus arvalis* 12.1% (95% CI = 5.0–23.3)
Genotype 30	*Apodemus flavicollis* 0.8% (95% CI = 0.0–4.3)	—
Genotype 34	*Clethrionomys glareolus* 0.8% (95% CI = 0.0–4.2)	—
Genotype 35	*Microtus arvalis* 1.4% (95% CI = 0.0–7.4)	*Apodemus flavicollis* 1.3% (95% CI = 0.2–4.5)
Genotype 36	—	*Apodemus agrarius* 3.5% (95% CI = 0.1–17.8)
Genotype 37	*Apodemus agrarius* 2.3% (95% CI = 0.1–12.0)	—
Genotype 38	*Apodemus flavicollis* 0.8% (95% CI = 0.0–4.3)	—
Genotype 39	*Clethrionomys glareolus* 0.8% (95% CI = 0.0–4.2)	—
Genotype 40	*Clethrionomys glareolus* 0.8% (95% CI = 0.0–4.2)	—
Genotype 41	*Clethrionomys glareolus* 0.8% (95% CI = 0.0–4.2)	—
Genotype 42	*Microtus arvalis* 1.4% (95% CI = 0.0–7.4)	—
Genotype 43	*Apodemus flavicollis* 0.8% (95% CI = 0.0–4.3)	—
Genotype 44	—	*Clethrionomys glareolus* 0.8% (95% CI = 0.0–4.2)
Genotype 45	—	*Microtus arvalis* 1.7% (95% CI = 0.0–9.2)
Genotype 46	—	*Apodemus flavicollis* 1.3% (95% CI = 0.6–3.5)
Genotype 47	—	*Apodemus flavicollis* 1.3% (95% CI = 0.6–3.5)
Genotype 48	—	*Clethrionomys glareolus* 0.8% (95% CI = 0.0–4.2)
Genotype 49	*Clethrionomys glareolus* 0.8% (95% CI = 0.0–4.2)	—
Genotype 50	*Apodemus flavicollis* 0.8% (95% CI = 0.0–4.3)	—

**Table 4:** The intraspecific genetic variability and neutrality test of *T*. *gondii* based on partial *ITS1* sequences.

Sample	*n*	*h*	*S*	*K*	*Hd* ± *SD*	*π* ± *SD*	Tajima *D*
Overall	239	51	63	0.63313	0.313 ± 0.036	0.00268 ± 0.00042	−2.80712 ***
Europe	132	19	22	0.36294	0.281 ± 0.052	0.00152 ± 0.00034	−2.58347 ***
Asia	68	24	38	1.88060	0.565 ± 0.074	0.00790 ± 0.00150	0.00790 ***
Americas	92	11	10	0.23865	0.167 ± 0.053	0.00101 ± 0.00038	−2.27301 **
Brazil	73	9	10	0.27397	0.184 ± 0.061	0.00115 ± 0.00046	−2.32423 **
China	9	1	0	0	0	0	*N/A*
Iraq	54	22	36	2.28372	0.631 ± 0.079	0.00956 ± 0.00179	−2.70545 ***
USA	18	3	2	0.11111	0.111 ± 0.096	0.00047 ± 0.00040	−1.16467
Overall Lithuania	127	19	22	0.37720	0.291 ± 0.054	0.00158 ± 0.00035	−2.58664 ***
*Apodemus agrarius* heart	5	2	1	0.400	0.400 ± 0.237	0.00167 ± 0.00099	−0.81650
*Apodemus agrarius* brain	5	2	1	0.400	0.400 ± 0.237	0.00167 ± 0.00099	−0.81650
*Apodemus flavicollis* heart	11	5	6	1.09091	0.618 ± 0.164	0.00456 ± 0.00187	−1.85059 *
*Apodemus flavicollis* brain	31	4	3	0.25376	0.243 ± 0.099	0.00106 ± 0.00046	−1.54377
*Clethrionomys glareolus* heart	30	6	6	0.40000	0.310 ± 0.109	0.00167 ± 0.00068	−2.09995 *
*Clethrionomys glareolus* brain	15	3	2	0.26667	0.257 ± 0.142	0.00112 ± 0.00064	−1.49051
*Microtus agrestis* heart	4	1	0	0	0	0	*N/A*
*Microtus arvalis* heart	14	3	2	0.28571	0.275 ± 0.148	0.00120 ± 0.00068	−1.48074
*Microtus arvalis* brain	15	3	2	0.26667	0.257 ± 0.142	0.00112 ± 0.00064	−1.49051
*Micromys minutus* heart	5	1	0	0	0	0	*N/A*

*n*—number of sequences, *h*—the number of genotypes, *S*—number of segregating sites, *K*—average number of nucleotide differences, *Hd*—haplotype diversity, *SD*—standard deviation, *π*—nucleotide diversity, *N/A*—not applicable, * *p* < 0.05, ** *p* < 0.01, *** *p* < 0.001.

## Data Availability

The partial *ITS1* sequences of *Toxoplasma gondii* were submitted to the NCBI GenBank database under accession numbers PX571996–PX572122.

## Supplementary Materials

The following supporting information can be downloaded at: https://www.mdpi.com/article/10.3390/pathogens14121252/s1, Table S1: *Toxoplasma gondii* prevalence in wild rodent species captured in Lithuania; Table S2: List of *T*. *gondii ITS1* region sequences from the NCBI GenBank used for analysis in this study; Table S3: Alignment of different *T*. *gondii ITS1* genotypes indicating nucleotide variations among sequences. Table S4: Information on wild rodents analyzed in this investigation.
